# Decentralized dynamic functional network connectivity: State analysis in collaborative settings

**DOI:** 10.1002/hbm.24986

**Published:** 2020-04-21

**Authors:** Bradley T. Baker, Eswar Damaraju, Rogers F. Silva, Sergey M. Plis, Vince D. Calhoun

**Affiliations:** ^1^ Tri‐Institutional Research Center in Neuroimaging and Data Science (TReNDS), Georgia State University, Georgia Institute of Technology, Emory University Atlanta Georgia USA; ^2^ Mind Research Network Albuquerque New Mexico USA; ^3^ University of New Mexico Albuquerque New Mexico USA

**Keywords:** brain imaging, data sharing, decentralized, decentralized algorithm, dFNC, dynamic functional network connectivity

## Abstract

As neuroimaging data increase in complexity and related analytical problems follow suite, more researchers are drawn to collaborative frameworks that leverage data sets from multiple data‐collection sites to balance out the complexity with an increased sample size. Although centralized data‐collection approaches have dominated the collaborative scene, a number of decentralized approaches—those that avoid gathering data at a shared central store—have grown in popularity. We expect the prevalence of decentralized approaches to continue as privacy risks and communication overhead become increasingly important for researchers. In this article, we develop, implement and evaluate a decentralized version of one such widely used tool: dynamic functional network connectivity. Our resulting algorithm, decentralized dynamic functional network connectivity (ddFNC), synthesizes a new, decentralized group independent component analysis algorithm (dgICA) with algorithms for decentralized *k*‐means clustering. We compare both individual decentralized components and the full resulting decentralized analysis pipeline against centralized counterparts on the same data, and show that both provide comparable performance. Additionally, we perform several experiments which evaluate the communication overhead and convergence behavior of various decentralization strategies and decentralized clustering algorithms. Our analysis indicates that ddFNC is a fine candidate for facilitating decentralized collaboration between neuroimaging researchers, and stands ready for the inclusion of privacy‐enabling modifications, such as differential privacy.

## Graphical abstract

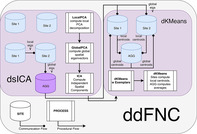

## 1 INTRODUCTION

The prospects of sharing data across studies provide researchers with clear and exciting prospects for collaborative analysis. Although the possible advantages to data‐sharing are clear—increasing sample size and diversity, for example—directly transferring samples between sites is not always feasible, or desirable. Lack of post hoc sharing provisions, tedious negotiations of data usage agreements (DUAs), and limitations on local storage and bandwidth may all impede efforts for direct sharing. Additionally, in privacy‐sensitive settings, direct sharing of data comes at the risk of reidentification, which becomes especially important in cases where samples belong to particularly rare groups, such as rare patient populations. Although steps toward anonymization in direct sharing scenarios can be taken, this anonymity often comes at the expense of data richness, or in the best cases, at the expense of significant effort by the collaborators involved.

Direct sharing of data is most often favored by centralized analysis frameworks, which pool data in one location. Though centralized sharing efforts can be powerful, to overcome the limitations outlined above, the research community requires a new family of decentralized approaches, where the analysis is performed without any direct data transfer, and data remains stored on disparate sites. One such decentralized alternative utilized by the ENIGMA framework performs meta‐analyses utilizing summary statistics and references to existing literature to perform analysis (Thompson et al., 2014, 2017). Though the approach ingeniously skirts issues endemic to centralized approaches, heterogeneity among studies and reliance on summary statistics tend to negatively impact the effectiveness of meta‐analysis approaches.

The answer to the shortcomings of meta‐analysis frameworks are iterative decentralized methods, where numerical optimization methods and other analysis techniques are split across multiple sites. Aggregation of shared iterates between sites allow these decentralized analysis frameworks to converge to solutions which are equivalent to the pooled case. The developers of the COINSTAC decentralized analysis framework (Plis et al., 2016) have successfully amassed a number of decentralized algorithms vital to neuroimaging analysis, including but not limited to independent vector analysis (Wojtalewicz, Silva, Calhoun, Sarwate, & Plis, 2017), deep neural networks (Lewis, Plis, & Calhoun, 2017), and voxel‐based morphometry (Gazula et al., 2018). In this work, we further one particular iterative pipeline, decentralized dynamic functional network connectivity (ddFNC), which combines a number of distinct and useful algorithms used primarily in neuroimaging analysis. We build on preliminary work introduced elsewhere (Gazula et al., 2018), extending the presentation of ddFNC to include more thorough analysis of the individual algorithms contained within it.

### 1.1 Dynamic functional network connectivity

Functional connectivity (FC) (Van Den Heuvel & Pol, 2010) is one popular method for neuroimaging analysis which evaluates the connectivity between functional networks extracted from functional magnetic resonance images (fMRI). In particular, the assessment of FC from resting‐state data has revealed new findings surrounding the high‐level spatio‐temporal organization of the brain. In this section, we present a framework for performing decentralized dynamic functional network connectivity (ddFNC) analysis (where FNC refers to the evaluation of FC between brain networks or components rather than isolated seeds). The resulting multistep framework includes decentralized versions for each step of the standard dynamic functional network connectivity (dFNC) pipeline, including novel algorithms for decentralized principal component analysis (GlobalPCA) and decentralized group independent component analysis (dgICA), as well as an application of decentralized K‐Means clustering to completely reproduce the full dFNC pipeline.

The standard, data‐driven approach to assess FNC dynamics, utilizes (a) spatial independent component analysis (ICA), (b) sliding window temporal correlation, and (c) k‐means clustering of windowed correlation matrices in order to evaluate connectivity between distinct functional networks. The approach, described by Allen et al. (2014) utilizes group ICA (GICA; Calhoun, Adali, Pearlson, & Pekar, 2001) to decompose resting‐state data from multiple subjects into statistically independent functional regions. To evaluate temporal dynamics in FNC, the correlation between component timecourses are then computed using a series of sliding windows (Sakoğlu et al., 2010). Finally, k‐means clustering is used to identify FNC patterns that reoccur in time and across subjects. These resulting clusters are called “FNC states,” describing short periods during which FNC topography remains relatively stable in the functional domain. In particular, these states and their shift over time can be used to evaluate group differences between patients suffering from various kinds of mental illness and healthy controls (Damaraju et al., 2014; Rashid, Damaraju, Pearlson, & Calhoun, 2014).

### 1.2 Federated learning for neuroimaging

Although no other methods for decentralized dFNC exist in the literature, a number of other approaches for federated learning on neuroimaging data exist in the literature. First, meta‐analysis frameworks such as ENIGMA (Thompson et al., 2014, 2017), perform analysis on local data, where meta‐statistics of the analyses are then aggregated in a decentralized fashion to produce global results. For example, Silva et al. implement the ENIGMA framework to provide structural analysis of subcortical brain data between multisite neuroimaging studies (Silva et al., 2019).

As mentioned above, meta‐analyses can introduce artifacts to standard machine‐learning algorithms due to heterogeneity between studies. As such, a number of approaches for iterative federated training of machine‐learning algorithms have been proposed in the literature. In general machine‐learning applications much focus has been given to federated deep learning (Bonawitz et al., 2019; Geyer, Klein, & Nabi, 2017; Konečnỳ et al., 2016; Konečnỳ, McMahan, Ramage, & Richtárik, 2016; Sattler, Wiedemann, Müller, & Samek, 2019; Smith, Chiang, Sanjabi, & Talwalkar, 2017), since training of deep learning models requires large amounts of data which may be decentralized across a data network.

In neuroimaging applications, a more diverse array of algorithms has recently appeared for federated learning. On the deep learning side, Lewis et al. propose apply a decentralized approach for deep learning to aid in the classification of neuroimaging addiction data (Lewis et al., 2017). Similarly, Remedios et al. provide a decentralized application of deep learning for neuroimage segmentation (Remedios et al., 2020). Decentralized joint independent component analysis (Baker, Silva, Calhoun, Sarwate, & Plis, 2015), independent vector analysis (Wojtalewicz et al., 2017), decentralized stochastic neighbor embeddings (Saha et al., 2019), and voxel‐based morphometry (Gazula et al., 2018) have also been applied to the analysis of decentralized neuroimaging data. In general, many of these frameworks proceed by iteratively computing the statistics used for optimization of a particular algorithm in a decentralized way. Although the statistics used for optimization are different and present novel challenges, our algorithm for ddFNC will proceed much in the same way.

## 2 MATERIALS AND METHODS

In this section, we present the data and experimental methodology utilized to evaluate decentralized group ICA, along with decentralized PCA (parallel and otherwise), decentralized clustering as well as the complete decentralized dFNC pipeline. First, Section 2.1 presents our novel method for performing group ICA in a decentralized setting. Second, Section 2.1.1 presents a novel method for performing decentralized PCA in parallel, improving the runtime of our previous decentralized PCA method.

Section 2.4 describes the functional MRI data used for evaluation of all novel methods. Then, Section 2.5 provides outlines of all the experiments performed for each method.

### 2.1 Decentralized group ICA

The first step in the dFNC pipeline for fMRI is group independent component analysis (gICA) (Calhoun et al., 2001). Suppose that sites collect data **X** ∈ ℝ^*d* × *N*^, where *d* is the size of the voxel dimension, and *N* is the total number of timepoints across all subjects on all sites. In linear spatial ICA, we model each individual subject as a mixture of *r* statistically independent spatial components, **A** ∈ ℝ^*d* × *r*^, and their timecourses, $S_{i}\in {\mathbb{R}}^{r\times N_{i}}$, where *N*_*i*_ is the length of the timecourse belonging to site *i*. Although there are multiple approaches to aggregating subjects for the group analysis (Rachakonda, Silva, Liu, & Calhoun, 2016), we can model the global (i.e., cross‐site) data set **X** as the column‐wise concatenation of *s* sites in the temporal dimension:

$$X=\left[{\mathrm{AS}}_{1}\cdots {\mathrm{AS}}_{i}\;\cdots \;{\mathrm{AS}}_{s}\right]\in {\mathbb{R}}^{d\times N},$$

where [⋯] represents column‐wise concatenation, *s* is the total number of sites in the consortium, and each site is modeled as a set of subjects concatenated in the temporal dimension as **AS**_*i*_ = **X**_*i*_ = [**X**_*i*1_⋯**X**_*im*_⋯**X**_*iM*_], that is, the collection of all *M* subjects in site *i*. The advantage of the temporal concatenation approach is that it only requires the computation of one ICA, yielding unique timecourses for each subject while assuming common group spatial maps. Thereafter, subject‐specific maps can be easily estimated via local back‐reconstruction. Spatial concatenation for group analysis is also possible, allowing for direct estimation of unique spatial maps while assuming common timecourses instead. Although the two approaches to concatenation amount to different ways of organizing the data, temporal concatenation appears to perform better for fMRI data (Schmithorst & Holland, 2004).

In this work, the goal is to learn a cross‐site global unmixing matrix, **B** ∈ ℝ^*N* × *r*^, such that $\hat{A}=\mathrm{XB}\approx A$, where $\hat{A}\in {\mathbb{R}}^{d\times r}$ is the set of unmixed maximally spatially independent components. To this end, we perform a decentralized group independent component analysis (dgICA), and use least squares to estimate the *m*‐th subject's temporal components in the *i*‐th site by computing ${\hat{S}}_{\mathrm{im}}=A^{-}X_{\mathrm{im}}$, where **A**^−^ is the pseudo‐inverse of the estimated sources.

Prior to ICA, we perform principal component analysis (PCA), as is typically done to reduce computational complexity and/or memory usage. In order to prevent disparate sites from obtaining full data samples, we resort to decentralized PCA (Baker et al., 2015). First, however, a (local) subject‐wise preprocessing step recommended prior to spatial GICA (Rachakonda et al., 2016) is performed, thus constituting a minor variation of the two‐stage decentralized PCA procedure utilized in Baker et al. (2015). Effectively, all sites preprocess each subject by removing local means in the voxel dimension, followed by reducing and whitening their temporal dimension to a common (and large) *k*_1_ components. Then, decentralized PCA of the preprocessed data takes place in the usual two stages. First, each site performs a LocalPCA dimension‐reduction (without whitening) of all preprocessed concatenated local subject data to a common *k*_2_ principal components in the temporal dimension. A decentralized second stage (GlobalPCA) then produces a global set of *r* spatial eigenvectors, **U** ∈ ℝ^*d* × *r*^. As outlined in Baker et al. (2015), this second stage asks sites to pass locally‐reduced eigenvectors to other sites in a round‐robin scheme where, upon receiving a set of eigenvectors, a site then stacks them in the column dimension along with its local preprocessed (but not *k*_2_ reduced) data, and performs a further reduction of the stacked matrix. The resulting (locally updated) set of *k*_2_ eigenvalues is then passed to the next peer in the network. This process iterates once through each site until the global eigenvectors reach some aggregator, or otherwise terminal site in the network. The algorithms for the LocalPCA and GlobalPCA steps are given in 3 and 1, respectively. Following the recommendation for choices of *k*_1_ and *k*_2_, we follow the recommendations in Erhardt et al. (2011) and Rachakonda et al. (2016), choosing *k*_1_ = 120 and *k*_2_ = 5 · *r*.

Algorithm 1 **Global PCA algorithm (GlobalPCA)**

**Require**: *s* sites with *preprocessed* data $\left\{X_{i}\in {\mathbb{R}}^{d\times k_{1}}:i=1,2,\ldots ,s\right\}$, intended final rank *r*, local rank 
*k* ≥ *r*.

1: Choose a random order *π* for the sites

2: 
**P**(1) = LocalPCA(**X**_
*π*(1)_, min{*k*, rank(**X**_
*π*(1)_)}) ⊳ Assume

3: **for** 
*j* = 2, 3, …, *s* **do** ⊳ Round‐robin scheme

4: 
*i* = **π**(*j*) ⊳ Set site index

5: Send 
**P**(*j* − 1) from site 
*π*(*j* − 1) to site 
*π*(*j*)


6: 
*k*^′^ = min{*k*, rank(**X**_
*i*_)}


7: 
**P**^′^ = LocalPCA(**X**_
*i*_, *k*^′^)


8: 
*k*^′^ = max{*k*^′^, rank(**P**(*j* − 1))}


9: 
**P**(*j*) = LocalPCA([**P**^′^ **P**(*j* − 1)], *k*^′^)


10: **end for**

11: 
*r*^′^ = min{*r*, rank(**P**(*s*))}


12: 
**U**= NormalizeTopColumns(
**P**(*s*), *r*^′^) ⊳ At last site

Algorithm 2 **NormalizeTopColumns**

**Require**: data 
**P** and number of columns to reduce 
*r*^′^
_._

1: $U=\left[P_{1}/\|P_{1}\|,P_{2}/\|P_{2}\|,\cdots ,P_{r^{\prime }}/\|P_{r^{\prime }}\|\right]$


  ⊳ first 
*r*^′^ columns of 
**P**


2: **return** U.

Algorithm 3 **Local PCA algorithm (LocalPCA)**

**Require**: data 
**X** ∈ ℝ^
*d* × *N*^ and intended rank *k*.

1: Compute the SVD 
**X** = **U**Σ**V**.

2: Let Σ^(*k*)^ ∈ ℝ^
*k* × *k*^ contain the largest *k* singular values and 
**U**^(*k*)^ ∈ ℝ^
*d* × *k*^ the corresponding singular vectors

3: Save 
**U**^(*k*)^ and Σ^(*k*)^ locally and return 
**P** = **U**^(*k*)^Σ^(*k*)^

After performing decentralized PCA either via GlobalPCA or some other decentralized algorithm, the aggregator site then performs whitening on these resulting global eigenvectors and runs a local ICA algorithm, such as infomax ICA (Bell & Sejnowski, 1995), or fastICA (Hyvarinen, 1999) to produce the spatial unmixing matrix, **W** ∈ ℝ^*r* × *r*^. The global eigenvectors, **U**, are then unmixed to produce $\hat{A}$ by computing $\hat{A}=\mathrm{UW}$, which is shared across the decentralized network (4). Each site *i* then uses this unmixing matrix to produce individual timecourses for each *m*‐th subject by computing ${\hat{S}}_{\mathrm{im}}=A^{-}X_{\mathrm{im}}$. Each site can then perform back‐reconstruction or spatio‐temporal regression (STR) approaches locally (Calhoun et al., 2001; Erhardt et al., 2011) to produce subject‐specific spatial maps, such as ${\hat{A}}_{\mathrm{im}}=X_{\mathrm{im}}S_{\mathrm{im}}^{-}$ in GICA1 back‐reconstruction, where $S_{\mathrm{im}}^{-}$ is the pseudo‐inverse of ${\hat{S}}_{\mathrm{im}}$.

Algorithm 4 **Decentralized group ICA algorithm (dgICA)**

**Require**: *s* sites with data $\left\{X_{i}\in {\mathbb{R}}^{d\times N_{i}}:i=1,2,\ldots ,s\right\}$, intended final rank *r*, local site rank 
*k*_2_ ≥ 5 · *r*, local subject rank 
*k*_1_ ≤ *N*_
*im*_.

1: **for all** sites 
*i* = 1, 2, …, *s* **do**

2: **for all** subjects 
*m* = 1, 2, …, *M* **do**

3:  $X_{i,m}^{\mathrm{pre}}=X_{i,m}-\mu \left(X_{i,m}\right)$


   ⊳ Remove **column** means.

4:  $X_{i,m}^{\mathrm{pre}}=$ NormalizeTopColumns(LocalPCA(**X**_
*i*,*m*_, *k*_1_), *k*_1_)

5: **end for**

6: **end for**

7: 
**U**= $\text{GlobalPCA}\left(\left\{X_{i}^{\mathrm{pre}}\in {\mathbb{R}}^{d\times k_{1}}:i=1,2,\ldots ,s\right\},r,k_{2}\right)$


8: 
**W** = ICA(**U**) ⊳ At aggregator site 
*i* = *π*(*s*)


9: Send $\hat{A}=\mathrm{UW}$ to sites 
*π*(1), …, *π*(*s* − 1)

Algorithm 5 **Back‐reconstruction**

**Require**: Global unmixing matrix $\hat{A}=\mathrm{UW}$, local data {*X*_
*i*,1_, …, *X*_
*i*,*M*_} on site *i*.

1: **for all** subjects 
*m* = 1, 2, …, *M* **do**

2: ${\hat{S}}_{i,m}={\hat{A}}^{-}X_{i,m}$ ⊳ Retrieve subject timecourses

3: ${\hat{A}}_{i,m}=X_{i,m}S_{i,m}^{-}$ ⊳ Use Spatio‐Temporal regression or other back‐reconstruction (Calhoun et al., 2001; Erhardt et al., 2011) to retrieve subject‐specific spatial maps

4: **end for**

#### 2.1.1 Parallel global PCA

**Figure 1:**
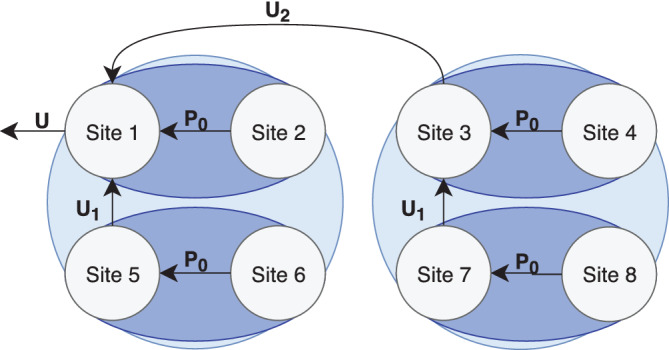
Diagram of the pGlobalPCA algorithm for a consortium of *s* = 8 sites, with cluster size *C* = 2. First, the recursion of the algorithm breaks the full consortium into clusters of decreasing size until the number of sites in each cluster is equal to *C*. Then, each cluster performs the standard GlobalPCA. As the recursion steps back from this base‐case, the result from GlobalPCA is passed between subclusters, and GlobalPCA performed again until the recursion ends

The global PCA algorithm given above in 1, taken from Baker et al. (2015)), can be extended from the serial version so that it runs in parallel, thus taking advantage of the decentralized nature of the computation to also increase computation speed. The parallel strategy involves breaking up the consortium into subclusters, where GlobalPCA is computed in parallel within the subclusters until the final eigenvectors *U* arrive at the aggregator. A diagram of the process for a consortium of eight sites is given in Figure 1, and the general algorithm is given in 6.

Algorithm 6 **Parallel Global PCA algorithm (pGlobalPCA)**

**Require**: *s* sites with data $\left\{X_{i}\in {\mathbb{R}}^{d\times N_{i}}:i=1,2,\ldots ,s\right\}$, intended final rank *r*, local rank 
*k* ≥ *r*, cluster size = *C*, base cluster size = *B*.

1: 
*K* = ⌊*s*/*C*⌋ ⊳ Number of Clusters

2: **if** *K* > *B* **then** ⊳ At an “aggregator” site

3: **for all** 
*c* = 1, 2, …, *K* **do**

4:  
*a* = (*c* − 1)*C* + 1


5:  
*b* = min(*c* · *C*, *s*)


6:  
**U**_
*c*_= pGlobalPCA(*b* – *a*, {**X**_
*a*_, …, **X**_
*b*_}, *k*, *r*)

7: **end for**

8: 
**U**= pGlobalPCA(*K*, {**P**_1_, …, **P**_
*K*_}, *k*, *r*)

9: **else** ⊳ at a non‐“aggregator” site

10: 
**U**= GlobalPCA(*s*, {*X*_1_, …, *X*_
*s*_}, *r*, *k*)

11: **if** At final aggregator **then**

12:  
**U** = NormalizeTopColumns(
**U**)

13: **end if**

14: **end if**

15: **return** 
**U**

### 2.2 Decentralized clustering

In order to perform dFNC in a decentralized setting, we first require a notion of decentralized clustering, used to cluster windowed patient timecourses into one of several connectivity states. Although other kinds of clustering are possible, previous work in dFNC has focused on the use of K‐Means clustering, and thus, we focus first on decentralized K‐Means clustering. A number of decentralized approaches to K‐Means exist: first, Dhillon and Modha (2002) implement an exact version of Lloyd's algorithm for K‐Means over distributed memory multiprocessors, where each processor broadcasts an updated set of local centroids, according to locally stored data, and global centroids are aggregated by taking the average of these local centroid updates. Jagannathan et al. implement a similar version of this approach, but add additional privacy guarantees via encrypted message passing, and random sharing of centroids, rather than sharing at each iteration (Jagannathan & Wright, 2005). Jagannathan et al. also provide a general version of privacy‐preserving clustering (including K‐Means), where rather than sharing centroid locations each iteration, local clusters are computed to convergence, and then merged at some aggregator site (Jagannathan, Pillaipakkamnatt, & Wright, 2006). Finally, a number of modern methods improve over standard methods with additional features often attractive in real‐network scenarios. For example, Datta et al. provide an approximative, peer to peer methods for distributed K‐Means (Datta, Giannella, & Kargupta, 2006; Datta, Giannella, & Kargupta, 2009), and Di et al. provide a fault‐tolerant version of dK‐Means, well‐suited to large, asynchronous networks (Di Fatta, Blasa, Cafiero, & Fortino, 2013).

**Figure 2:**
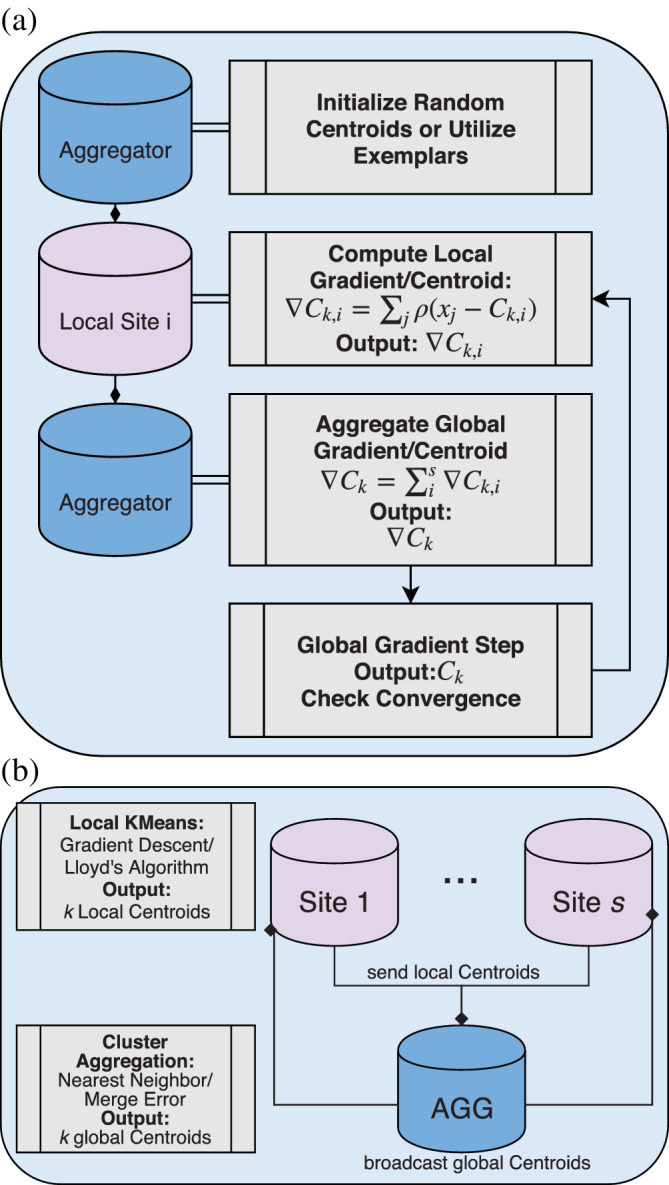
Diagram of the multishot and single‐shot dK ‐ Means algorithms. Panel a outlines the multishot schema using gradient descent or Lloyd's algorithm. First, randomized centroids are picked by the aggregator, and broadcast out to the sites. Each site then computes cluster membership, and perform their dK ‐ Means updates, either by computing a gradient, or by updating the centroid according to Lloyd's algorithm. These are then broadcast back to the aggregator, and aggregated into new centroids or gradients. New centroids are then rebroadcast, and the algorithm continues until convergence. In panel b, a diagram of the single‐shot schema is given. In this approach, each site performs a separate, local K‐Means optimization, and the final centroids are broadcast to the aggregator, which then merges clusters either by merging nearest centroids, or by querying sites to compute a merging error, as is done in Jagannathan et al. (2006)

Our aim in this article is to provide a novel, end‐to‐end pipeline for decentralized dFNC, which includes clustering. Thus, which exact choice of algorithm is made for the decentralized K‐Means step is an implementation choice, rather than an essential part of our pipeline. For our purposes, we test four different version of simple decentralized K‐Means algorithms, focusing primarily on differences in centroid computation and updates, rather than details such as asynchronous updates, or peer to peer schema. First, we implement the algorithm from Dhillon and Modha (2002), and we also implement a version of the same iterative algorithm using a decentralized gradient update, rather than exact centroid computation. For this latter strategy, we implement the gradient‐descent algorithm described in Bottou and Bengio (1995), where at each iteration, locally computed gradients are averaged on the aggregator node in place of locally computed centroids. Finally, we implement version of these algorithms using the cluster aggregation strategy described in Jagannathan et al. (2006); however, we omit the additional privatization strategies for simplicity's sake. The two former strategies we call “multishot,” because they involve decentralization at each iteration of the algorithm, and the two later strategies we call “single‐shot” because they involve aggregation of the results of locally converged optimization strategies (Figure 2).

To perform clustering for distributed dFNC, we first have each site compute sliding‐window timecourse correlations for each subject, where the window length is fixed across the decentralized network. Additionally, initial clustering is performed on a subset of windows from each subject, corresponding to windows of maximal variability in correlation across component pairs. To obtain these exemplars, we follow the approach from Damaraju et al. (2014), and have each site compute variance of dynamic connectivity across all pairs of components at each window. We then select windows corresponding to local maxima in this variance timecourse. This results in an average of eight exemplar windows per subject. We then perform decentralized K‐Means on the exemplars to obtain a set of centroids, which are shared across the decentralized network, which we feed into a second stage of K‐Means clustering.

**Figure 3:**
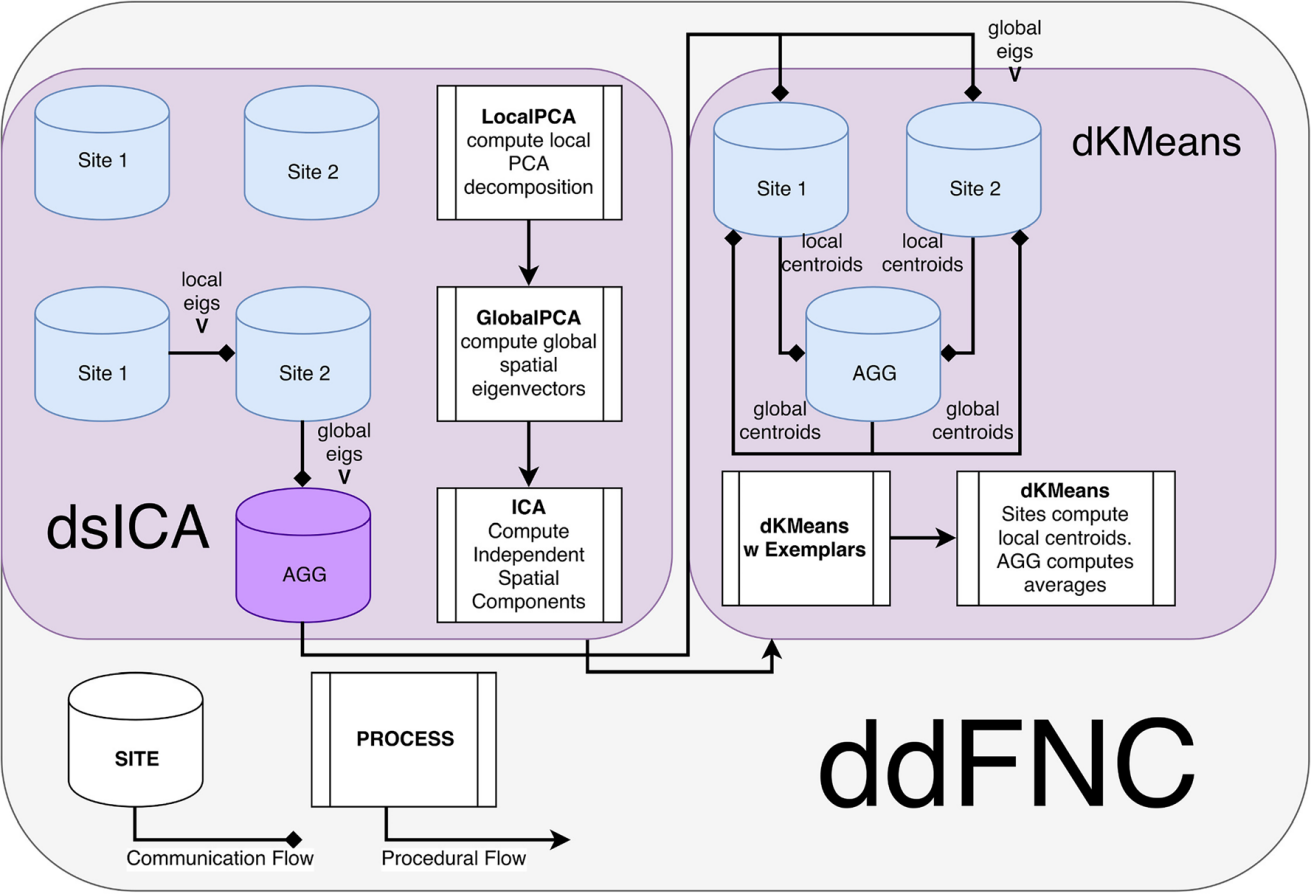
Flowchart of the ddFNC procedure, for example, with two sites, using multishot Lloyd's algorithm for K‐Means clustering. To perform dgICA, sites first locally compute subject‐specific LocalPCA to reduce the temporal dimension, and then use the GlobalPCA procedure from Baker et al. (2015) to compute global spatial eigenvectors, which are then sent to the aggregator. The aggregator then performs ICA on the global spatial eigenvectors, using InfoMax ICA (Bell & Sejnowski, 1995), for example, and passes the resulting spatial components back to local sites. The dK‐Means procedure then iteratively computes global centroids using the procedure outlined in Dhillon and Modha (2002), first computing centroids from subject exemplar dFNC windows, and then using these centroids to initialize clustering over all subject windows

For the second stage of decentralized clustering, at each iteration, each site computes updated centroids according to Dhillon and Modha (2002), which corresponds to a local K‐Means update. These local centroids are then sent to the aggregator node, which computes the weighted average of these updated centroids, and rebroadcasts the updated global centroids until convergence. A summary of the complete steps in the dFNC pipeline is given in Figure 3.

Algorithm 7 **Decentralized dFNC algorithm (ddFNC)**

**Require**: *s* sites with data $\left\{X_{i}\in {\mathbb{R}}^{d\times N_{i}}:i=1,2,\ldots ,s\right\}$, win‐size *t*, number of clusters *k*.

1: dgICA → **W**, global unmixing matrix. Compute $\hat{A}=\mathrm{UW}$, and broadcast to sites

2: **for all** sites = 
*i* = 1, 2, …, *s* **do**

3: ${\hat{S}}_{i,m}={\hat{A}}^{-}X_{i,m}$  ⊳ Back‐reconstruct subject TCs

4: **for all** windows 
*w* = 1, 2, …, *N*_
*i*_ − *t* **do**

5:  ${\hat{S}}_{i,m,w}=\left[{\hat{S}}_{i,m,w}\ldots {\hat{S}}_{i,m,\left(w+t\right)}\right]$ ⊳ Sliding window of size *t* over time

6:  $V_{i,m,w}=\text{corr}\left({\hat{S}}_{i,m,w}\right)$


7: **end for**

8: Obtain local exemplar correlation matrices 
**V**_
*i*, *ex*_ using the process from Damaraju et al. (2014)

9: **end for**

10: Run dK‐Means(
**V**_
*ex*_) (Dhillon & Modha, 2002) to obtain *k* initial centroids, 
**C**_0_


11: Run dK‐Means(
**V**) (Dhillon & Modha, 2002) with initial clusters 
**C**_0_ to obtain *k* centroids 
**C**, and clustering assignment for each instance, *L*

12: Return 
**C**, *L*

### 2.3 Computational complexity

Because ddFNC is a pipeline containing multiple distinct algorithmic components, the overall computational complexity of the pipeline will depend greatly on implementation details for each pipeline stage. The choice of ICA algorithm, or whether or not an iterative method is used to computed SVD, for example, will greatly influence the actual complexity of the entire pipeline. That said, we provide an initial analysis of the GlobalPCA component of our pipeline as presented here, with the caveat that further changes can still be made within each of these depending on implementation preferences and availability of computational resources. We omit an analysis of complexity for ICA, since in principle any ICA algorithm could be used, and complexity varies with the choice of algorithm.

The overall computational complexity of ddFNC is best analyzed in terms of the complexity on individual sites, since the decentralization of the algorithm reduces overall complexity into a sum of individual computational demands at each site. Suppose at an individual site, we begin with the matrix of temporally concatenated subjects $X_{i}\in {\mathbb{R}}^{d\times N_{i}}$.

The complexity of Global PCA can be analyzed in terms of the complexity for the two singular value decompositions performed first on the covariance matrix $X_{i}\in {\mathbb{R}}^{N_{i}\times N_{i}}$, and second on the *k*_1_ × *k*_1_ matrix computed from stacking eigenvectors. Standard algorithms for computing SVD by Jacobi rotations have a complexity of *O*(*n*^3^) when computed on an *n* × *n* covariance matrix (Cline & Dhillon, 2006). Thus, if GlobalPCA uses standard SVD, the overall complexity will be $O\left(N_{i}^{3}\right)+O\left(k_{1}^{3}\right)$, with complexity generally increasing as the number of subjects on local sites increases, or as the desired number of independent components increases.

Prior to decentralized K‐Means, each site computes correlation matrices on the windowed timecourses of length *N*_*i*, *j*_ − *w* for each subject *j*. If *m*_*i*_ subjects are located at a given site, then the local complexity for computing these matrices is $O\left(m_{i}\left(N_{i}-w\right)k_{1}^{3}\right)$, so again local computational cost increases with the number of subjects, the number of timepoints at each subject, and the desired number of independent components *k*_1_.

For an analysis of decentralized K‐Means, we refer the reader to the discussion in Dhillon and Modha (2002). Let J is the number of K‐Means iterations required for the centroid stability for K‐Means with *C* centroids, and let *S*_*i*_ = *m*_*i*_(*N*_*i*_ − *w*) be the number of correlation matrices computed at each site. The analysis provided in Dhillon et al. gives the site‐wise computational complexity for dK‐Means as $O\left(\left(3{\mathrm{Ck}}_{1}^{2}+S_{i}C+S_{i}k_{1}^{2}+{\mathrm{Ck}}_{1}^{2}\right)\cdot J\right)$. For our pipeline, the choice of decentralized K‐Means algorithm is modular, and local site complexity may be reduced in a number of different ways. For example, implementing a decentralized version of K‐Means++ initialization (Arthur & Vassilvitskii, 2006; Bachem, Lucic, Hassani, & Krause, 2016; Bahmani, Moseley, Vattani, Kumar, & Vassilvitskii, 2012) to may lower the number of iterations required for stability, thus reducing site‐wise complexity as well as overall complexity. Because further analysis requires digging into the particulars of K‐Means and decentralized K‐Means which is outside the scope of this article, we leave further analysis of complexity for K‐Means as future work.

### 2.4 Functional MRI data for dFNC

To evaluate ddFNC, we utilize imaging data from Damaraju et al. (2014) collected from 163 healthy controls (117 males, 46 females; mean age 36.9) and 151 age—and gender matched patients with schizophrenia (114 males, 37 females; mean age 37.8), for a total of 314 subjects.

The scans were collected during an eyes closed resting fMRI protocol at seven different sites across the United States (see Table 1) and pass data quality control. Informed and written consent was obtained from each participant prior to scanning in accordance with the Internal Review Boards of corresponding institutions (Potkin & Ford, 2008). A total of 162 brain‐volumes of echo planar imaging BOLD fMRI data were collected with a temporal resolution of 2 s on 3‐Tesla scanners.

**Table 1:** Distribution of subjects over original seven sites

Site	HC	SZ	Total
1	28	24	52
2	9	9	18
3	28	26	54
4	28	23	51
5	14	13	27
6	28	29	57
7	28	27	55

Imaging data for six of the seven sites was collected on a 3 T Siemens Tim Trio System and on a 3 T General Electric Discovery MR750 scanner at one site. Resting‐state fMRI scans were acquired using a standard gradient‐echo echo planar imaging paradigm: FOV of 220 × 220 mm (64 × 64 matrix), TR = 2 s, TE = 30 ms, FA = 770, 162 volumes, 32 sequential ascending axial slices of 4 mm thickness and 1 mm skip. Subjects had their eyes closed during the resting‐state scan. Data preprocessing for dgICA was performed according to the preprocessing steps in Damaraju et al. (2014).

### 2.5 Experiments

In this section, we describe each of the experiments performed to step the various parts of our ddFNC pipeline. Since the ultimate goal is to provide ddFNC, we concentrate the bulk of our quality analysis on that final output; however, at each stage, we perform a number of small evaluations to make sure each piece works individually using either simulated data. We also measure the runtime of each stage separately, and compare runtimes and quality measures for different implementations of each algorithm.

#### 2.5.1 Decentralized group ICA

In this section, we present the experimental methodology used to evaluate decentralized group ICA, which includes decentralized PCA.

##### Do pGlobalPCA and GlobalPCA produce equivalent components?

**Figure 4:**
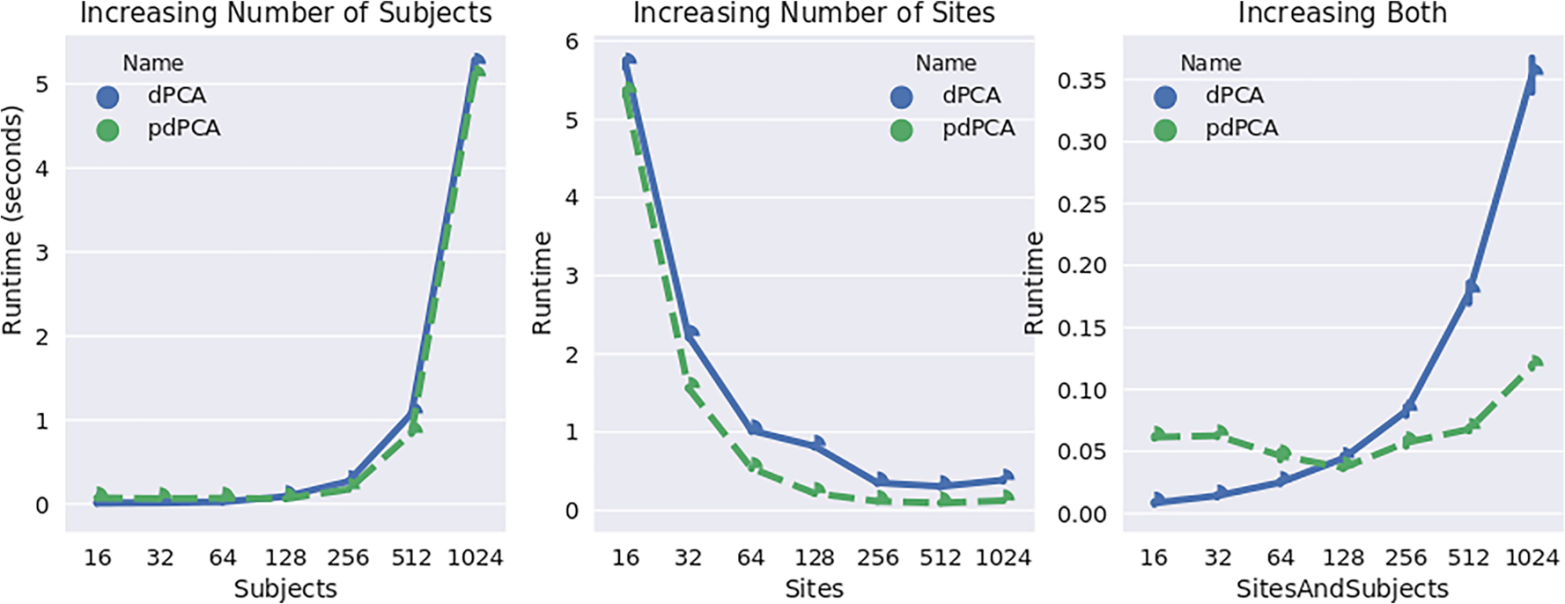
Runtime comparison of GlobalPCA (1) and Parallel Global PCA (pGlobalPCA, 6) for three different scenarios. In panel (a), we increase the number of subjects in a global consortium with two fixed sites. In panel (b), we increase the number of sites in a global consortium, keeping the number of subjects fixed at 1,024. In panel (c), we increase the number of sites and subjects simultaneously. The blue curve represents the mean runtime over 10 repeated runs for the GlobalPCA algorithm, and the green curve represents the mean runtime over 10 repeated runs for the pGlobalPCA algorithm

Although it is clear mathematically that pGlobalPCA and GlobalPCA are equivalent, we perform a brief initial experiment to provide empirical evidence of the equivalence. First, to evaluate our novel method for parallel decentralized PCA, we generate a synthetic data set using the MATLAB randn function. We generate a single 100 × 100 data set, and use pooled PCA, GlobalPCA, and pGlobalPCA to reduce the column dimension to 10 principal components. For GlobalPCA and pGlobalPCA, we first split the data set onto 10 simulated “sites,” where each site contains 10 rows of the original matrix. If pGlobalPCA and PCA are functionally equivalent, we expect the correlation matrices to be nearly completely diagonal. We repeat this experiment 1,000 times for each algorithm, and plot the results in Figure 4.

**Figure 5:**
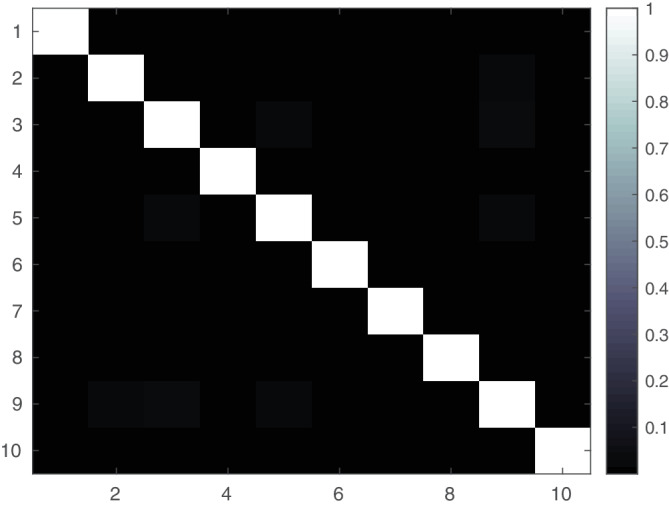
Correlation of components estimated from GlobalPCA and Parallel GlobalPCA, averaged over 10 separate runs

After completing the synthetic experiments, we perform the same experiment using the real data described above. We utilize the site distribution used above, and again compute the correlation of the estimated PCs, and plot the results in Figure 5. For real data, we repeat each experiment 100 times, with each repetition shuffling subjects between the sites.

##### How does pGlobalPCA improve runtime?

The parallelization in pGlobalPCA (6) should provide a performance improvement over vanilla GlobalPCA (1), especially in consortia with a large number of sites, allowing for many computations to be performed in parallel. In order to test this hypothesis, we perform two experiments designed to evaluate the runtime of GlobalPCA and pGlobalPCA, and how pGlobalPCA offers an improvement over GlobalPCA for certain distributions of subjects over the network.

First, we perform an experiment with synthetic data, using the same data‐generation process as above. In order to evaluate how the runtime improvement for pGlobalPCA varies depending on the subject/site distribution, we vary both the size of the global data set and the number of sites in the consortium in order to evaluate how the distribution of data affects the runtime of both algorithms.

Again, we repeat a similar experiment utilizing the real data set, evaluating how the distribution of subjects over the network affects the runtime of GlobalPCA and pGlobalPCA. We begin with two subjects, and increase by powers of two until we are dividing the 314 subjects over 64 sites.

##### How does the choice of ICA method affect performance?

ddFNC is a highly modular algorithm, thus allowing for the aggregator node in a given consortium to choose from any kind of Group ICA algorithm made available. Thus, we perform a brief analysis which compares multiple ICA algorithms in terms of component estimation quality and runtime. To measure the quality of components, we match the estimated components from the given ICA algorithm with the components estimated in Damaraju et al. (2014), selecting the top components which best match with that ground truth. Then, we compute the Moreau–Amari Inter‐Symbol Interference index (Amari, Cichocki, & Yang, 1996) between the estimated components and the components from Damaraju et al. (2014), and plot the results for the given choice of algorithm. We note that in Damaraju et al. (2014), the authors utilize infomax ICA, and so a decentralized infomax will have a comparative edge over other methods.

#### 2.5.2 Decentralized clustering

We perform dK‐Means (Dhillon & Modha, 2002) on the computed correlation matrices from the sliding windows described above. We first cluster the “exemplar” temporal windows computed for each subject according to the strategy utilized in Damaraju et al. (2014), and then utilize these centroids to cluster the entire set of computed windows. This provides a set of a *k* = 5 resulting centroids as well as clustering assignments for each subject's window.

#### 2.5.3 Decentralized dFNC

In this section, we present the experimental methodology used to evaluate the final results of the decentralized dynamic dFNC pipeline.

We verify that ddFNC can generate sensible dFNC clusters by replicating the centroids produced in Damaraju et al. (2014). We closely follow the experimental procedure in Damaraju et al. (2014), with some of the additional postprocessing omitted for simplicity. To evaluate the success of our pipeline, we run a simple experiment where we implement the ddFNC pipeline end‐to‐end on the data, simulating 314 subjects being evenly shared over two decentralized sites.

We use a window length of 22 timepoints (44 s), for a total of 140 windows per subject. For dgICA, we first estimate 120 subject‐specific principal components locally, and reduce each subject to 120 points in the temporal dimension. Subjects are then concatenated temporally on each site, and we use the parallel GlobalPCA algorithm to estimate 100 spatial components, and perform whitening. We then use local infomax ICA (Bell & Sejnowski, 1995) on the aggregator to estimate the unmixing matrix **W**, and estimate 100 spatially independent components, $\hat{A}$. We then broadcast $\hat{A}$ back to the local sites, and each site computes subject‐specific timecourses.

After spatial ICA, we have each site perform a set of additional postprocessing steps prior to decentralized dFNC. First, we select 47 components from the initial 100, by computing components which are most highly correlated with the components from Damaraju et al. (2014). We then have each site drop the first two points from each subject, regress subject head movement parameters with six rigid body estimates, their derivatives and squares (total of 24 parameters). Additionally, any spikes identified are interpolated using third order spline fits to good neighboring data, where spikes are defined as any points exceeding mean (FD) + 2.5 * std(FD), where FD is framewise displacement (interpolating 0–9 points (mean, *SD*: 3, 1.76)).

For clustering in general, elbow‐criterion estimation can be used to determine an optimal number of clusters. For comparison's sake, however, we use the optimal number of clusters from Damaraju et al. (2014), setting *k* = 5. For the exemplar stage of clustering, we evaluate 200 runs where we initialize centroids uniformly randomly from local data, and then run dK‐Means using the cluster averaging strategy in Dhillon and Modha (2002). For our distance measure, we use scikit‐learn (Pedregosa et al., 2011) to compute the correlation distance between covariance matrices following the methods in Damaraju et al. (2014). To keep our implementation simple, unlike Damaraju et al. (2014), we do not utilize graphical LASSO to estimate the covariance matrix, and thus do not optimize for any regularization parameters. Additionally, we do not perform additional Fisher‐Z transformations or perform additional regularization using a previously computed static dFNC result. Future implementations may also utilize a decentralized sFNC algorithm as preprocessing, as is done for the pooled case in Damaraju et al. (2014). Finally, for the second stage of dK‐Means, we initialize using the centroids from the run with the highest silhouette score, computed using the scikit‐learn python toolbox (Pedregosa et al., 2011), again running dK‐Means to convergence. After computing the centroids, we use the correlation distance and the Hungarian matching algorithm (Kuhn, 1991) to match both plotted spatial components from dgICA and the resulting centroids from dK‐Means.

Finally, to make a more direct comparison between our analysis and the pooled case, we compare the resulting centroids with centroids estimated using pooled K‐Means, measuring the correlation between the resulting centroids over multiple runs.

We also separate out the centroids for each group, and visualize them according to the procedures in Damaraju et al. (2014). Following the procedures in Damaraju et al. (2014), we first calculated the element‐wise subject medians for each state according to the final clustering assignments from dK‐Means. We then use the subject medians for each state and evaluated the differences between patient and healthy‐control groups using a two sample *t*‐test.

## 3 RESULTS AND DISCUSSION

### 3.1 GlobalPCA versus pGlobalPCA

In Figure 5, we plot the correlation of the components estimated from GlobalPCA and pGlobalPCA, averaged over 10 repeated runs, where each run created a new simulated matrix to be reduced. Clearly, the results indicate near‐equivalence of the two algorithms, with minor differences likely due to noise from the serial GlobalPCA utilizing a different, random ordering of sites, or from the stochastic nature of infomax ICA.

In Figure 4, we plot the average runtime for GlobalPCA and pGlobalPCA across three different scenarios of changing the subject and site distributions across a consortium. In panel a, we increase the number of subjects in a global consortium with two fixed sites. In panel b, we increase the number of sites in a global consortium, keeping the number of subjects fixed at 1,024. In panel c, we increase the number of sites and subjects simultaneously.

The runtime comparison for the fixed number of sites in panel a illustrate the equivalent runtime for each algorithm in a scenario where the total number of sites is equal to the number of allowed cluster groups in pGlobalPCA. In such cases, where the parameter *b* is set to equal the number of sites in the consortium, pGlobalPCA is equivalent to GlobalPCA, and the algorithms perform comparatively.

The runtime comparison in figure b, however, illustrates the benefits of parallel, decentralized PCA. In a highly distributed setting, where the number of sites is much larger than the parameter *b*, pGlobalPCA decreases runtime over standard GlobalPCA by executing certain steps in parallel, leveraging the decentralized design to improve runtime.

Panel c illustrates both a failure case for pGlobalPCA, where increased bandwidth between many small sites with small data invokes a small hit in runtime; however, it also illustrates that pGlobalPCA does not suffer as significant of a hit as more sites are added into the consortium, whereas the serial design of GlobalPCA suffers significantly.

### 3.2 dgICA results

**Figure 6:**
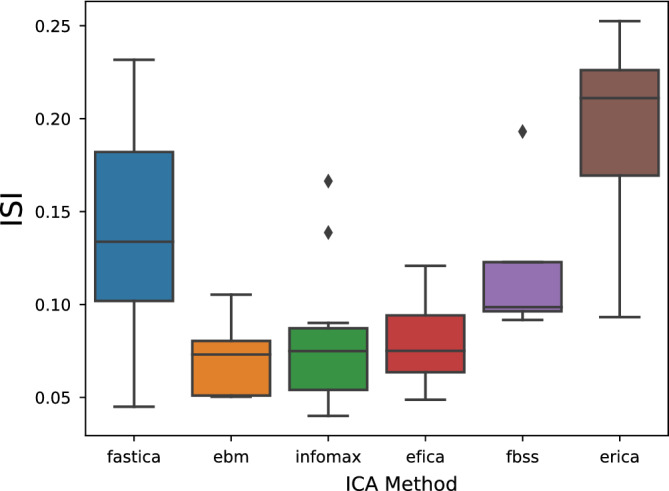
The Moreau–Amari Index (*y*‐axis) computed for our algorithm, compared over multiple ICA algorithms (*x*‐axis). Choices of ICA algorithm were evaluated 10 times over the same set of principal components, and then compared with the ground truth set of estimated components

**Figure 7:**
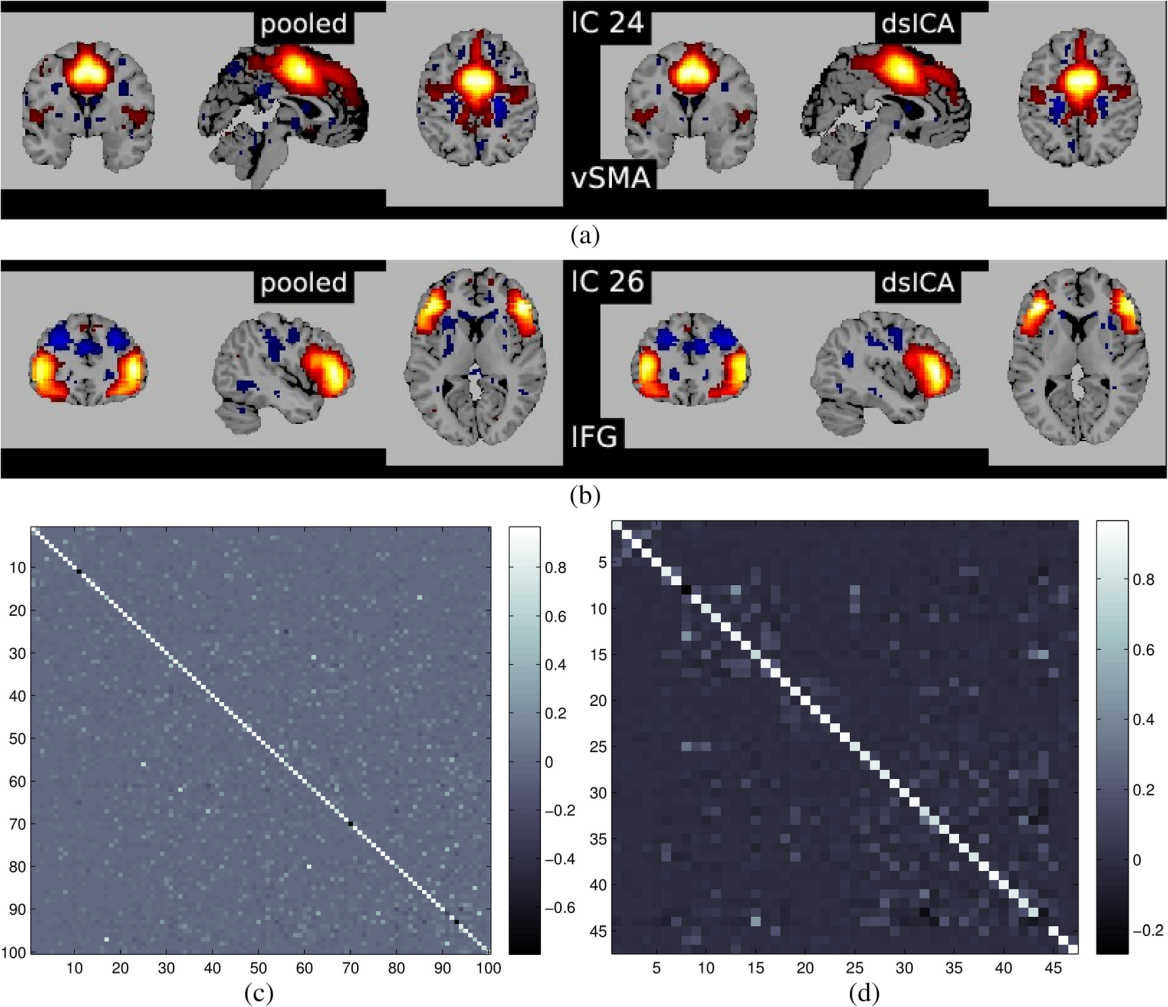
Panels (a) and (b) illustrate examples of matched spatial maps from dgICA and pooled ICA. Panels (c) and (d) show the correlation of the components between pooled spatial ICA and dgICA after Hungarian matching. Panel (c) shows correlation between all 100 components, and panel (d) shows correlation between the 47 neurological components selected in Damaraju et al. (2014)

Figure 6 plots the Moreau–Amari Index for several different ICA algorithms performed at the aggregator node. In Figure 7 we plot some of the estimated components from dgICA with infomax ICA, and compare with the matched components from pooled ICA. We also provide the correlation of estimated components in Figure 7c,d. Indeed, dgICA with Infomax ICA provides components which are a good estimate of the pooled case, with the ISI between pooled and decentralized cases measured below 0.1, and the component‐wise correlation of components providing a near exact estimate of the pooled components. This indicates that our decentralization strategy works, and does not incur a significant penalty to quality of the estimations via decentralization alone. This assurance of quality in decentralization may change when privacy measures, such as differential privacy, are taken; however, our analyses here is sufficient to show that decentralization alone does not significantly affect the quality of estimation, and we leave the further problem of assuring estimation along with quality for future work.

### 3.3 ddFNC results

In Figure 7, we plot some examples of the components estimated from decentralized spatial ICA in comparison with the spatial components from Damaraju et al. (2014), after performing Hungarian matching between the estimated spatial maps. We also plot the correlation of the components from our ICA implementation in comparison to the components from Damaraju et al. (2014). Indeed, the estimated components are highly correlated with the results from Damaraju et al. (2014), for all 100 estimated components, as well for the 47 selected neurological components from Damaraju et al. (2014), indicating that dgICA is able to produce results comparable to the pooled case. We include additional spatial maps for all 47 estimated spatial components in the Supporting Information.

**Figure 8:**
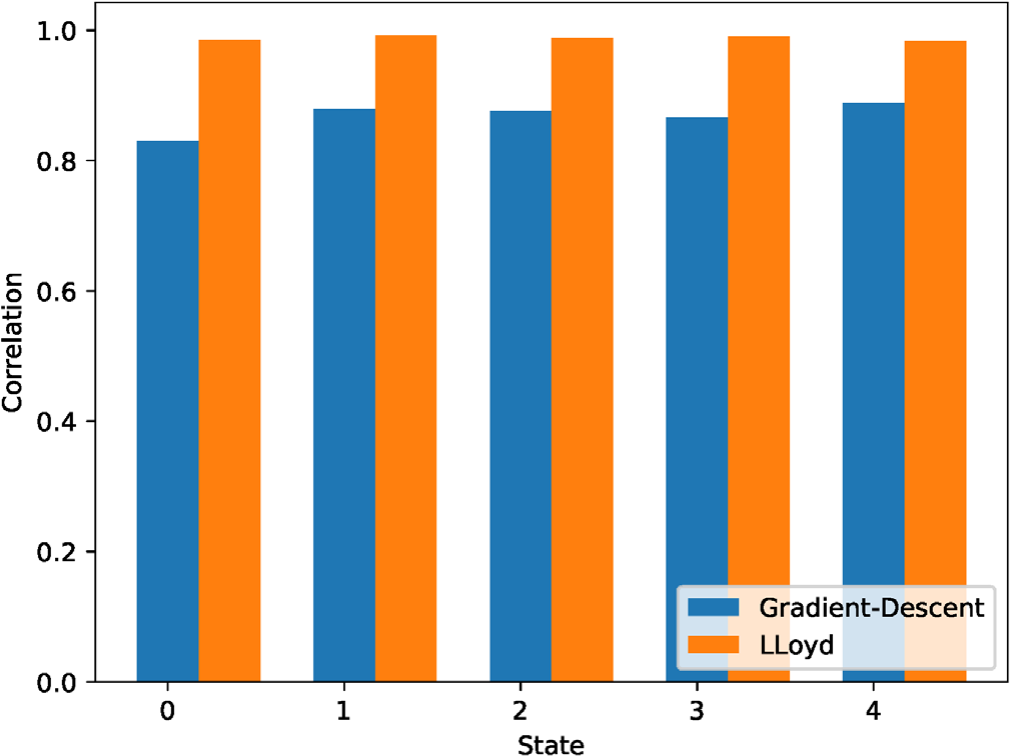
Correlation between pooled centroids and decentralized centroids estimated using decentralized Lloyd's algorithm and decentralized gradient descent. The centroids from Lloyd's algorithm are much closer to the pooled case

First, in Figure 8, we plot the correlation between the centroids estimated with our method, and those estimated with a pooled gICA and pooled K‐Means. Decentralized centroids estimated with decentralized Lloyd's algorithm match better to the pooled case, with each centroid correlating above 98% with the pooled estimation. The gradient‐descent implementation does not converge to the pooled solution as well, though the results are still greatly similar, correlating above 85% with the pooled case.

The improved performance of decentralized Lloyd's algorithm can be explained in part by the lack of thorough hyper‐parameter searching for the gradient‐descent‐based algorithm, which would likely improve the results. For the purpose of this work, since Lloyd's algorithm provides near perfect estimation of the pooled centroids, we leave the task of decentralized hyper‐parameter searching for future work.

**Figure 9:**
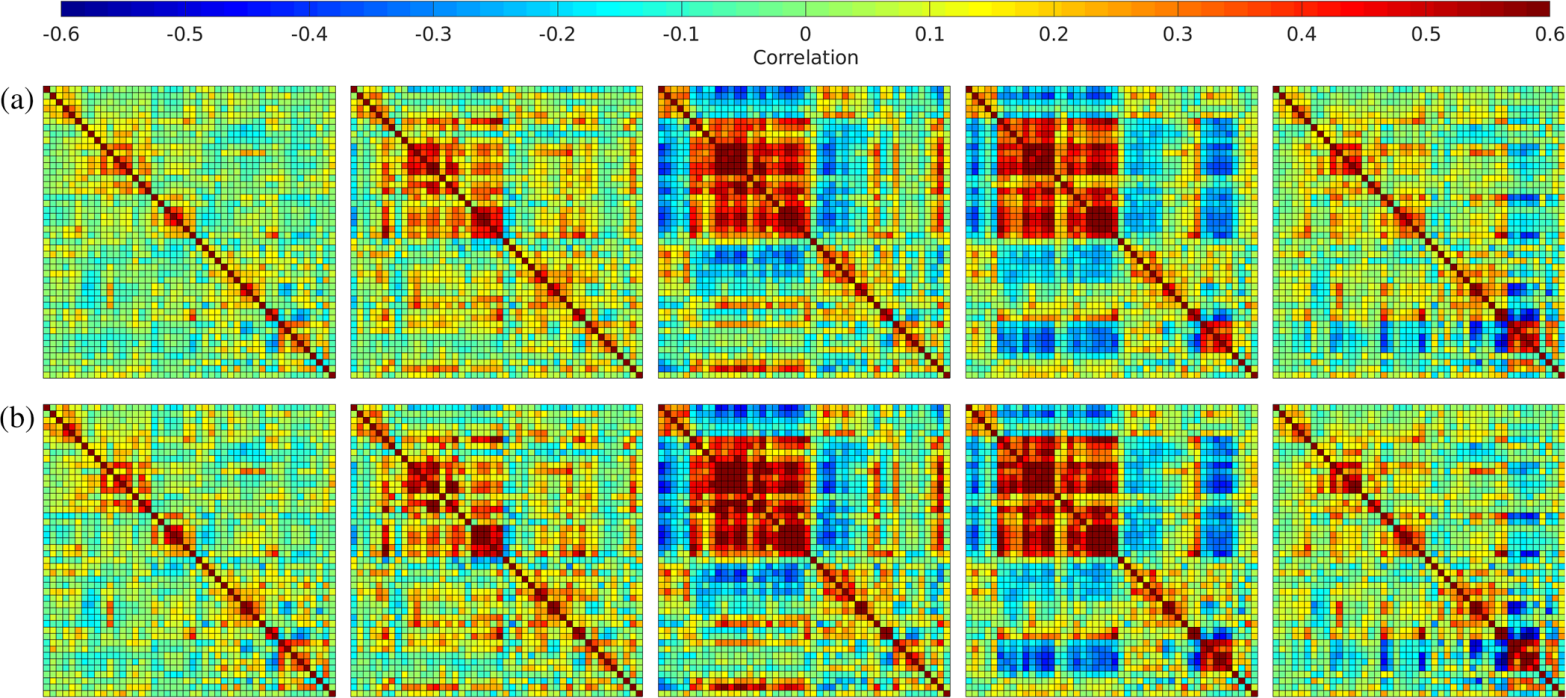
The *k* = 5 median centroids over all groups for pooled dFNC from Damaraju et al. (2014) (panel a), and the hungarian‐matched centroids from ddFNC (panel b)

In Figure 9, we plot the centroids from Damaraju et al. (2014) (panel a), as well as the centroids estimated using decentralized dFNC (panel b). Additionally, we plot the correlation between the centroids estimated with our method, and those estimated in the pooled case, given in Figure 8.

**Figure 10:**
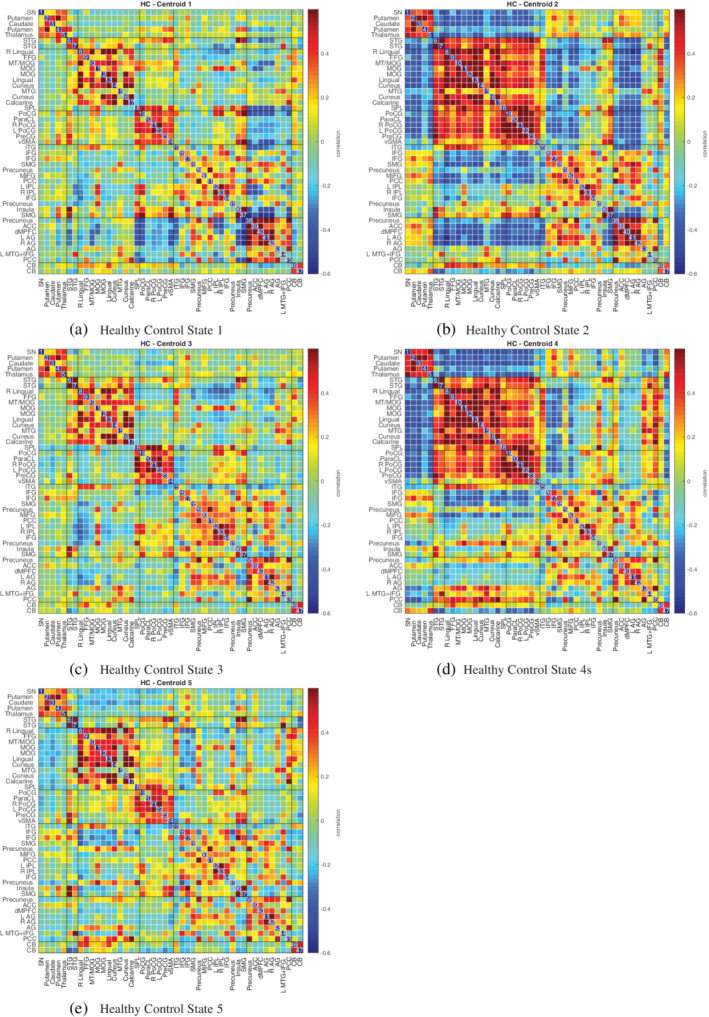
Estimated median states for 163 healthy controls, computed using decentralized dFNC with *k* = 5, using the original site configuration from the Fbirn data set described in Section 2.4

**Figure 11:**
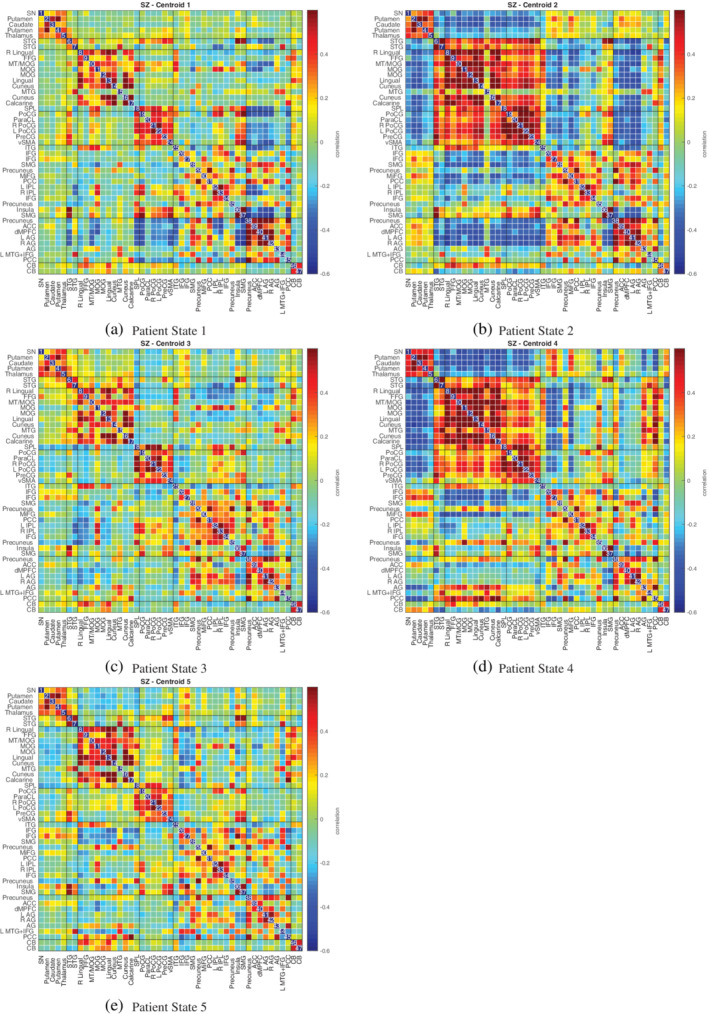
Estimated median states for 151 patients, computed using decentralized dFNC with *k* = 5, using the original site configuration from the Fbirn data set described in Section 2.4

**Figure 12:**
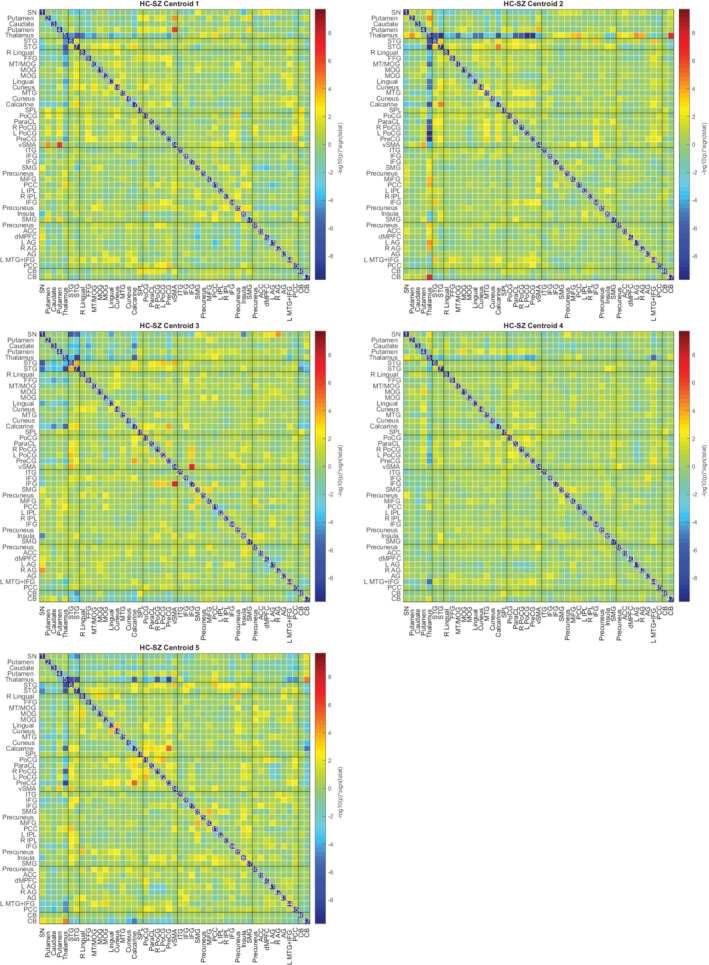
Estimated median group differences for a two‐tailed *t*‐test between the 151 patients and 163 healthy controls, computed after decentralized dFNC with *k* = 5, using the original site configuration from the Fbirn data set described in Section 2.4

In Figures 10, 11, 12, we plot the group centroids for healthy controls (Figure 10), patients with schizophrenia (Figure 11), and the differences between each group (Figure 12). Although our results show slight differences compared to the analysis in Damaraju et al. (2014), States 2 and 4 from our estimation closely resemble States 2 and 3 in Damaraju et al. (2014), with the high anticorrealtion within the sensory and motor regions. Our estimation of State 4 best fits with State 3 from Damaraju et al. (2014), showing greater sensory‐motor anticorrelations than our State 2, as well as higher activation in the default mode. Our States 1 and 5 bear striking similarity to one another, and best compare with States 4 and 5 from Damaraju et al. (2014), while our State 3 compared best with State 1 from Damaraju et al. (2014).

### 3.4 Privacy

One of the advantages of decentralized analysis pipelines is that only intermediary statistics are passed between sites, and full patient records never are released across the network. These kinds of decentralized algorithms are “plausibly private” (Sarwate, Plis, Turner, Arbabshirani, & Calhoun, 2014), due to the lack of directly identifiable records in the global data network. Our pipeline for ddFNC is clearly plausibly private, since no full data instances are explicitly passed between sites during analysis.

The limitation of plausibly private algorithms is that the actual ensured privacy is not quantifiable, with risk of identification never clearly assured. Measures such as Cynthia Dwork's differential privacy (Dwork, 2008) have been proposed to alleviate the concerns of plausible privacy, with concrete mechanisms available to ensure privacy up to a given level with some loss of model utility accrued in exchange for privacy assurances (Dwork & Roth, 2014).

The addition of differential privacy introduces further problems to a pipeline which often involve new variables in the pipeline such as optimal privacy mechanism, choice of privacy budget, and how the privatized algorithm compares in terms of utility with nonprivatized models. Thus, our pipeline represents an important first step toward fully differentially private ddFNC, providing a clear direction for future work.

## 4 CONCLUSION

In this article, we presented a simple case study of how functional network connectivity analysis can be performed on multisite data without the need for pooling data at a central site. The study shows that both the decentralized regression as well as the decentralized dynamic functional network connectivity yield results that are comparable to its pooled counterparts guaranteeing a virtual pooled analysis effect by a chain of computation and communication process. Other advantages of such a decentralized platform include data privacy and support for large data. Further extensions to the decentralized regression algorithm presented here include: adding a regularization term (ridge, lasso and elastic‐net) to the objective function, standardized development of gradient‐descent schemes to perform optimization in a more iterative fashion and developing a differential privacy version for each algorithm. In conclusion, the results presented here strongly encourage the use of decentralized algorithms in large neuroimaging studies over systems that are optimized for large‐scale centralized data processing.

## CONFLICT OF INTEREST

This work was supported by grants from the NIH grant numbers R01DA040487, P20GM103472, and R01EB020407 as well as NSF grants 1539067 and 1631838. The authors declare that there was no other financial support or compensation that could be perceived as constituting a potential conflict of interest.

## Data Availability

The source code for decentralized dynamic functional network connectivity (ddFNC) is available online as a part of the Collaborative Information and Neuroimaging Suite Toolkit for Anonymous Computation (COINSTAC). The full source for COINSTAC can be found at the URL https://github.com/MRN-Code/coinstac, and the source code for ddFNC in particular can be found at the URL https://github.com/MRN-Code/coinstac_ddfnc_pipeline. The functional magnetic resonance imaging data utilized for analysis is from the Functional Biomedical Research Network (FBIRN) Data Repository. This data is not available for release due to IRB restrictions; however, derivatives of the data may be available upon request.
